# Evaluation of CONSRANK-Like Scoring Functions for Rescoring Ensembles of Protein–Protein Docking Poses

**DOI:** 10.3389/fmolb.2020.559005

**Published:** 2020-10-21

**Authors:** Guillaume Launay, Masahito Ohue, Julia Prieto Santero, Yuri Matsuzaki, Cécile Hilpert, Nobuyuki Uchikoga, Takanori Hayashi, Juliette Martin

**Affiliations:** ^1^CNRS, UMR 5086 Molecular Microbiology and Structural Biochemistry, University of Lyon, Lyon, France; ^2^Department of Computer Science, School of Computing, Tokyo Institute of Technology, Tokyo, Japan; ^3^Tokyo Tech Academy for Leadership, Tokyo Institute of Technology, Tokyo, Japan; ^4^Department of Network Design, School of Interdisciplinary Mathematical Sciences, Meiji University, Tokyo, Japan

**Keywords:** protein–protein interaction, docking, scoring, prediction, interface

## Abstract

Scoring is a challenging step in protein–protein docking, where typically thousands of solutions are generated. In this study, we ought to investigate the contribution of consensus-rescoring, as introduced by Oliva et al. (2013) with the CONSRANK method, where the set of solutions is used to build statistics in order to identify recurrent solutions. We explore several ways to perform consensus-based rescoring on the ZDOCK decoy set for Benchmark 4. We show that the information of the interface size is critical for successful rescoring in this context, but that consensus rescoring in itself performs less well than traditional physics-based evaluation. The results of physics-based and consensus-based rescoring are partially overlapping, supporting the use of a combination of these approaches.

## Introduction

Protein–protein docking aims at predicting the structure of a complex starting from the structures of isolated components (Melquiond et al., 2012; Vakser, 2014). The CAPRI community-wide initiative allows a blind assessment of the participant methods on common data sets and evaluation criteria, offering an updated view of progress in the field since 2001 (Lensink et al., 2007, 2017; Lensink and Wodak, 2010). Protein–protein docking methods typically generate thousands of potential solutions for a particular complex. Scoring the models to discriminate near-native solutions is a known bottleneck of docking methods (Moal et al., 2013a,b; Malhotra et al., 2015). Most scoring functions are physics-based, attempting to capture the determinants underlying the stability of protein–protein complexes, e.g., shape complementary, electrostatics and desolvation potential (Dominguez et al., 2003; Cheng et al., 2007; Pierce and Weng, 2007, 2008; Moal and Bates, 2010; Ritchie and Venkatraman, 2010; Ohue et al., 2014). Knowledge-based functions, on the other hand, aim at taking advantage of the information from available structures, *via* pair potentials (Lu et al., 2003; Huang and Zou, 2008; Mezei, 2017), or multibody potentials (Khashan et al., 2012). Docking methods often use scoring functions that combine physical terms with knowledge-based terms (Kozakov et al., 2006; Liang et al., 2009; Feliu et al., 2011; Vreven et al., 2011). More recently, evolutionary information has been successfully used for scoring (Andreani et al., 2013; Yu et al., 2017).

Another approach consists in relying on the recurrences observed in the set of solutions, i.e., consensus-based scoring. Consensus-based scoring functions seek to identify solutions with features that are the most frequent in the solution set, independently of any physics-based or evolutionary evaluation. The CONSRANK scoring function, proposed by Oliva et al. (2013, 2015), Vangone et al. (2013), and Chermak et al. (2015, 2016) has shown very good results, based on the conservation of interface contacts.

In this study, we compare several CONSRANK-like scoring functions on large sets of docking poses generated by ZDOCK, including CONSRANK. We then explore how to combine consensus-based rescoring with the native scoring function of ZDOCK.

## Methods

### Docking Decoy Set

The ZDOCK3.0.2 decoy set (Pierce et al., 2011) (6 degree sampling, fixed receptor format) for Benchmark4 (Hwang et al., 2010a) was retrieved from https://zlab.umassmed.edu/zdock/decoys.shtml. This data set encompasses 176 protein–protein complexes, with 54,000 docking poses for each complex. For each pose, the interface Cα RMSD, with respect to the bound structure, is given. A near-native docking hit is defined as a prediction with interface Cα RMSD < 2.5 Å.

### Consensus-Based Rescoring Schemes

Following the CONSRANK method (Oliva et al., 2013; Chermak et al., 2015), docking poses are rescored using the frequencies of interface contacts in the set of docking poses. Interface contacts are defined using a distance cut-off of 5 Å between the heavy atoms of receptor and ligand proteins.

For each contact *C*_*ij*_ between residue *i* from receptor and residue *j* from ligand the relative frequency in the decoy set is defined by:

(1) $$S\left(C_{ij}\right)=\frac{F\left(C_{ij}\right)}{N}\in \left[0,1\right]$$

where *F*(*C*_*ij*_) denotes the frequency of *C*_*ij*_ at the protein–protein interface in the set of *N* decoys. These relative frequencies are then averaged, i.e., normalized by the interface size, to compute the CONSRANK score of each pose *P*:

(2) $$CONSRANK\_score\left(P\right)=\frac{\sum_{C_{ij}\in P}S\left(C_{ij}\right)}{N_{\text{cont}}\left(P\right)},$$

where *N*_cont_(*P*) denotes the number of interface contacts in docking pose *P*.

### Variations of CONSRANK Scores

First, we considered the un-normalized version of CONSRANK scores (Oliva et al., 2013), denoted as CONSRANK_U, where the relative frequencies of interface contacts are only summed, and not averaged:

(3) $$CONSRANK\_U\left(P\right)=\sum_{C_{ij}\in P}S\left(C_{ij}\right).$$

Then, we implemented two other variations, by replacing relative frequencies of contacts *S*(*C*_*ij*_) by relative frequencies of residues:

(4) $$S\left(R_{i}\right)=\frac{F\left(R_{i}\right)}{N}\in \left[0,1\right]$$

where *F*(*R*_*i*_) denotes the frequency of residue i at the protein–protein interface (distance between heavy atoms lower than 5 Å) in the set of *N* decoys. The two related scores are respectively defined by:

(5) $$Residue\_Average\left(P\right)=\frac{\sum_{R_{i}\in P}S\left(R_{i}\right)}{N_{res}\left(P\right)},$$

(6) $$Residue\_Sum\left(P\right)=\sum_{R_{i}\in P}S\left(R_{i}\right).$$

where *N*_*res*_(*P*) denotes the number of interface residues in pose *P*. Here, interface residues are simply those involved in contacts at the interface.

Note that is it possible to compute the contact and residue frequencies (Eqs 1 and 4) on a given set of docking poses and then to evaluate another set of docking poses (with Eqs 2, 3, 5, and 6).

### Clustering

We implemented the BSAS clustering procedure (Basic Sequential Algorithmic Scheme) (Koutroumbas and Theodoridis, 2008; Jiménez-García et al., 2018) to reduce the structural redundancy of docking poses. The principle of BSAS is the following. Docking poses are ranked according to a score in decreasing order. The pose with the highest score initiates the first clusters. The other poses are sequentially compared to already clustered poses: they are included in a cluster if they are within a given cut-off of cluster members, otherwise they initiate a new cluster. At the end of the process, the pose with the highest score in each cluster is the representative of each cluster. In order to allow a fast clustering process, we do not compute the RMSD between ligand atoms. Instead we use a distance cut-off between the centers of mass of the ligands, here set to 8 Å.

### Evaluation

The top 2,000 solutions according to the ZDOCK native scoring function were rescored using the rescoring schemes detailed below. We monitored the presence of near-native docking hits (interface Cα RMSD < 2.5 Å) in the top 10 solutions after re-ranking. Each protein–protein complex with a near-native docking hit in the top 10 solutions is counted as a success.

### Implementation

The consensus-based rescoring functions are implemented in python code accessible on GitHub, which operates directly on ZDOCK output files, and allows to treat rapidly thousands of docking poses (typically a few seconds for 2,000 poses, up to 1 min for 54,000 poses). In addition, the code allows to compute statistics on a given set of poses and re-score another of structures (see “Results” section). All the scripts necessary to reproduce the results shown in this article are available at: https://github.com/MMSB-MOBI/CHOKO.

## Results

In this study, we compare four consensus-based scores to identify the near-native solutions among the ensembles generated by ZDOCK. The traditional CONSRANK score (Oliva et al., 2013) is considered, as well as its un-normalized version, and two variations that consider residue statistics instead of contact statistics. We first evaluate each consensus score separately. Then, we combine these results with the native ZDOCK physics-based scoring function. Finally, we add a clustering step, to reduce structural redundancy and further improve the results.

### Quality of Decoys

In the initial data set of 176 protein–protein complexes, ZDOCK was able to generate at least one near-native docking hit (interface Cα RMSD < 2.5 Å) in the first top 2,000 solutions for 90 protein–protein complexes. These 90 protein–protein complexes thus constitute our reference data set for the rest of the study. We explore if and how consensus-based rescoring is efficient at scoring the decoys of these 90 protein–protein complexes.

### Evaluation of Different Consensus-Based Rescoring Functions

First, we compare the four versions of consensus-based rescoring functions: either contact-based [following the CONSRANK (Oliva et al., 2013) scheme] or residue-based, with or without interface size normalization. We estimate the performance by counting the number of successes, i.e., number of complexes with at least one near-native hit (interface Cα RMSD < 2.5 Å) in the first 10 solutions after rescoring. We also tested the effect of varying the subset of docking poses used to compute the contact and residue frequencies (Eqs 1 and 4): we used either the first 50, 100, 1,000 or 2,000 first poses provided by ZDOCK, or the full set of 54,000 poses, referred as the frequency set. In any case, we rescored the first 2,000 poses provided by ZDOCK.

**FIGURE 1:**
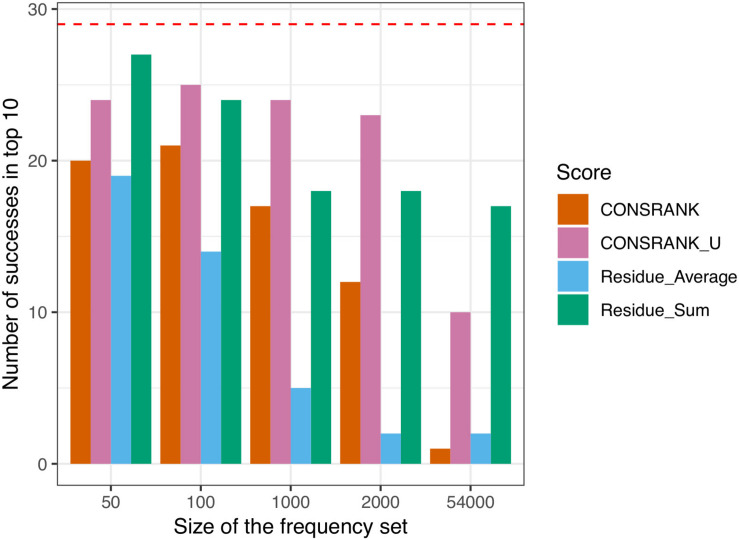
Number of successes after rescoring the first 2,000 solutions of ZDOCK. The size of the frequency set refers to the set of poses used to compute the residue and contact scores from Eqs 1 and 4. The horizontal red dashed line indicates the number of successes achieved by the ZDOCK native scoring function.

The results of this evaluation are shown in Figure 1. We can see that un-normalized rescoring functions (CONSRANK_U and Residue_Sum) constantly outperform the normalized rescoring functions (CONSRANK, Residue_Average). The size of the subset used to compute contact and residue frequencies (Eqs 1 and 4) has a major influence on the number of successes. Indeed, ZDOCK solutions are ranked by the ZDOCK native scoring function; hence the top of the list is, in many cases, enriched in near-native docking hits. Estimating contact and residue scores on a reduced subset of poses at the top of the list is logically more efficient. On the contrary, estimating contact and residue scores from the full list leads to a loss of information, and worsens the prediction. When frequencies were estimated on the 54,000 poses, the number of successes was 1 for CONSRANK, 10 for CONSRANK_U, 2 for Residue_Average, and 17 for Residue_Sum. In the best settings tested here, estimating the scores on the first 50 solutions to rescore the first 2,000 solutions allows to reach a number of successes equal to 27 with the Residue_Sum scoring function, *versus* 20 for the CONSRANK scheme. It is thus possible to rescore large sets of docking poses using consensus-based scoring functions, with better performance than the commonly used CONSRANK scheme.

### Combination With ZDOCK Native Scoring Function

In this section, we explore how to combine rescoring functions with the native scoring function of ZDOCK. Out of the 90 protein–protein complexes with at least one near-native docking hit in the top 2,000 solutions, the ZDOCK native scoring function identifies 29 successes, i.e., 29 complexes with at least one near-native docking hit in the top 10, see Figure 1. This is indeed better than the four consensus-based rescoring functions tested here. One could wonder if it is then possible to improve the initial prediction of ZDOCK using rescoring.

We first analyzed the overlap of the successful cases by ZDOCK and each rescoring function, and found that many successful cases achieved by rescoring are well predicted by the ZDOCK scoring function, see Supplementary Figure S1. For example, when estimating scores on the first 50 solutions for the rescoring (Supplementary Figure S1A), all the successes identified by the normalized rescoring functions CONSRANK and Residue_Average are included in the successes identified by ZDOCK. Un-normalized scoring functions are able to identify 1 case not included in the ZDOCK successes for CONSRANK_U, and 4 for Residue_Sum.

We then tested a combination of ZDOCK poses and rescored poses by combining the first *N*_*1*_ poses of ZDOCK with the first *N*_*2*_ poses after rescoring, with *N*_*1*_ + *N*_*2*_ = 10, and no redundancy. Again, we vary the subset of docking poses used to compute the contact and residue frequencies with Eqs 1 and 4 (frequency set = top 50, 100, 1,000 or 2,000 poses) and in any case, we rescore the first 2,000 solutions provided by ZDOCK using Eqs 2, 3, 5, and 6. We estimate the performance by counting the number of successes, i.e., number of complexes with at least one near-native docking hit (interface Cα RMSD < 2.5 Å) in the first 10 solutions.

The results of this evaluation are shown in Supplementary Figure S2. Regardless of the size of the frequency set, the best combination is always obtained with the Residue_Sum scoring function. Combining the first six ZDOCK poses with the first four Residue_Sum rescored poses, and estimating the frequencies on the full set of 2,000 poses (bottom right panel in Supplementary Figure S2) allows to reach a number of successes equal to 32, compared to 18 with Residue_Sum alone and 29 with ZDOCK alone. This suggests the possibility to marginally improve the native results of ZDOCK by a simple combination of poses. It is interesting to note that, in this situation, the information about residues is more efficient in rescoring than the information about pairwise contacts.

### Combining Clusters

**FIGURE 2:**
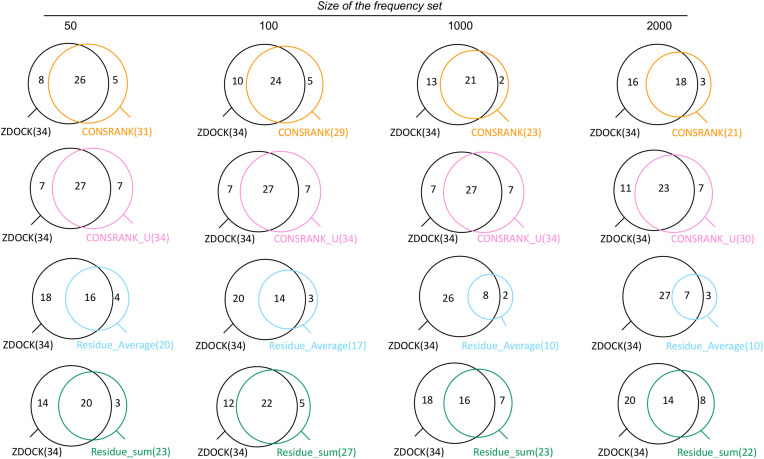
Venn diagrams showing the overlap between successful cases with the ZDOCK native scoring function and each of the rescoring functions, when using structural clustering.

Clustering is classically used to improve the performance, by reducing the structural redundancy of docking solutions (Kozakov et al., 2005; Hwang et al., 2010b; Koukos et al., 2020). Here, we used the BSAS clustering algorithm, which takes into account the scores, to cluster poses by their ligand center of mass. When applied to ZDOCK results, independently of rescoring, we obtained an improvement in terms of number of successes: 34 successes instead of 29, reflecting a structural redundancy of the ZDOCK set. We first analyzed the overlap between ZDOCK results and the results of each rescoring function when using structural clustering, see Figure 2.

As shown in Figure 2, the results of ZDOCK and rescoring functions are partially overlapping also after structural clustering. The rescoring functions are able to identify between 2 and 8 additional successful cases, with more additional cases brought by un-normalized scoring functions CONSRANK_U and Residue_Sum. This means that a perfect combination of ZDOCK and rescoring with no loss would reach a number of successes equal to 42.

We then explore how to combine ZDOCK results and rescoring results. We have tested a combination of clusters. On the one hand, we computed clusters from the poses ranked by their initial ZDOCK scores. On the other hand, we computed clusters from poses reordered after consensus rescoring. We then combine the representative poses of the first *N*_*1*_ ZDOCK clusters, with the representative poses of the first *N*_*2*_ poses after rescoring, with *N*_*1*_ + *N*_*2*_ = 10. We estimate the performance by counting the number of successes, i.e., number of complexes with at least one near-native hit (interface Cα RMSD < 2.5 Å) in the first 10 solutions.

**FIGURE 3:**
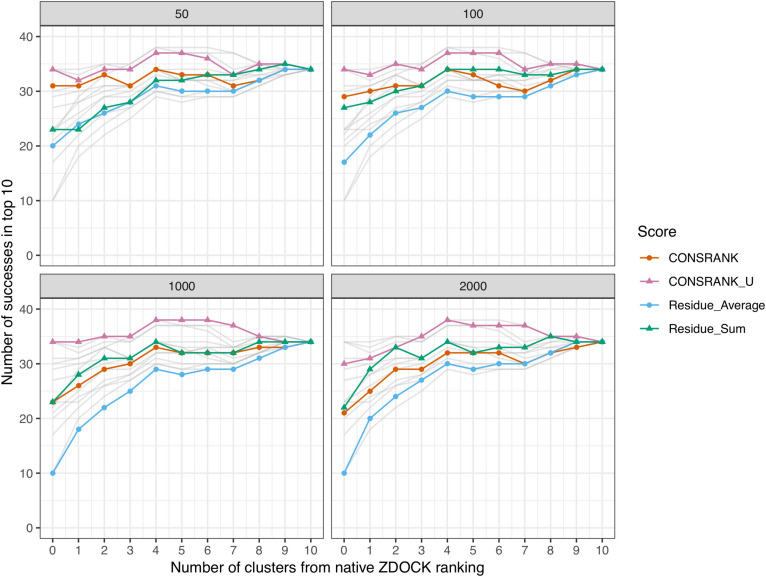
Number of successes after combination of clusters with the ZDOCK native scoring function. Each panel corresponds to different frequency sets, i.e., sets of poses used to compute the residue and contact scores from Eqs 1 and 4. In any cases, the first 2,000 solutions of ZDOCK are rescored. Gray lines represent data from other panels for comparison.

The results of this evaluation are shown in Figure 3. In agreement with the Venn diagram analysis, the best combination is obtained using an un-normalized rescoring function, CONSRANK_U. It constantly outperforms CONSRANK, regardless of the frequency set. When using the first 1,000 poses to estimate the frequencies (bottom left panel in Figure 3), the combination of five ZDOCK clusters and five CONSRANK_U clusters achieves a number of successes equal to 38, compared to 34 with ZDOCK alone and 34 with CONSRANK_U alone. This result suggests that CONSRANK-like rescoring could be used together with physics-based evaluation. Contrary to what was observed in simple pose combination (Figure 2), the most efficient rescoring scheme when dealing with clusters is based on contact frequencies, not residue frequencies. It seems that, after structural clustering, the information of pairwise contacts, which is more precise than residues, becomes more useful in discrimination.

### Illustrative Examples

**FIGURE 4:**
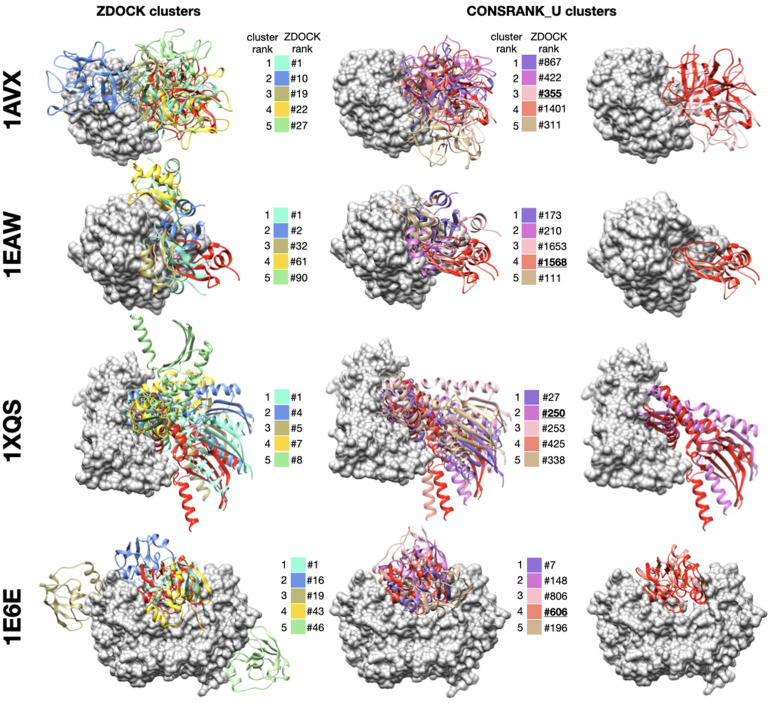
Examples of successful combination of ZDOCK clusters and consensus-based clusters. For each protein–protein complex, the receptor protein is represented as a gray surface and the ligand as a red ribbon. Left column: representative poses of the first five clusters generated with ZDOCK native scoring function, middle column: representative poses of the first five clusters generated with the CONSRANK_U rescoring function, right column: superimposition between near-native docking hit and native structure. For each representative pose, initial ZDOCK rank is indicated next to the color legend. Near-native poses are underlined. 1AVX (Song and Suh, 1998): complex between the porcine trypsin (gray) and soybean inhibitor (red), 1EAW (Friedrich et al., 2002): complex between the catalytic domain of serine proteinase MT-SP1 (gray) and bovine inhibitor (red), 1XQS (Shomura et al., 2005): complex between the human Hsp70 binding protein 1 (gray) and Hsp70 (red), 1E6E (Müller et al., 2001): complex between NADPH:adrenodoxin oxidoreductase (gray) and adrenoxin (red).

To complete this study, in this section, we present examples to illustrate the asset of consensus rescoring when used in combination with the native ZDOCK scoring function. We used the results generated using the CONSRANK_U function, with frequencies estimated on the first 1,000 poses, and combined five ZDOCK clusters and five consensus clusters. As explained in the previous section, this setting allows to reach 38 successes. We present four examples from the ZDOCK decoy set where the use of consensus rescoring is critical in Figure 4. For all these protein–protein complexes, no near-native docking hit is observed in the first 10 clusters of ZDOCK (or in the first 10 poses). The use of CONSRANK_U rescoring in conjunction with clustering allows the identification of near-native docking poses in the top 10. In every case, these near-native poses do not belong to the top of the ZDOCK initial list: they are ranked 355 for 1AVX, 1568 for 1EAW, 250 for 1XQS, and 606 for 1E6E. These examples highlight the usefulness of consensus-based rescoring to rescue poses with poor initial ranks.

For the 38 successful complexes in this experiment, we systematically computed the number of near-native poses coming from ZDOCK clusters and the number of near-native poses coming from CONSRANK_U clusters. Detailed results are provided in Supplementary Table S1 for the 38 complexes with at least one near-native pose in the top ten. In eight cases, the near-native poses were present only in ZDOCK clusters, in 10 cases, the near-native poses were present only in CONSRANK_U clusters and in the 20 remaining cases, near-native poses were present in both ZDOCK and CONSRANK_U clusters. We observed no significant bias in terms of functional category or interface size between protein complexes that were successful only with ZDOCK or only with CONSRANK_U. This indicates that ZDOCK and CONSRANK_U results are only partially overlapping, justifying the need to combine them.

## Conclusion

We have implemented four variants of consensus-based rescoring functions: the CONSRANK score, the CONSRANK un-normalized score, and their equivalents based on residue frequencies and tested them on the rescoring of large sets of docking poses of the ZDOCK benchmark. In this context, un-normalized scores that do take into account the size of the interfaces are in general more efficient than normalized scores. When used alone, consensus-based scoring functions degraded the initial performance of the physics-based ZDOCK scoring function. However, when both physics-based and consensus-based scoring functions were used in combination, we observed a marginal improvement. This calls for calibration when using consensus-based scoring functions to re-rank large sets of docking decoys, since they are, by definition, highly dependent on the docking decoy population.

## Data Availability Statement

All datasets presented in this study are included in the article/Supplementary Material.

## Author Contributions

GL, JS, CH, and JM contributed to software. GL, YM, and NU contributed to investigation. MO and JM contributed to the methodology. TH contributed to the data curation. JM conceptualized and wrote the manuscript. All authors contributed to the article and approved the submitted version.

## Conflict of Interest

The authors declare that the research was conducted in the absence of any commercial or financial relationships that could be construed as a potential conflict of interest.
