# Supplementary material for: Evaluation of CONSRANK-Like Scoring Functions for Rescoring Ensembles of Protein–Protein Docking Poses
Source: Front Mol Biosci. 2020 Oct 21;7:559005. doi: 10.3389/fmolb.2020.559005 (PMC7641601; doi:10.3389/fmolb.2020.559005)
Supplement: Supplementary file 1 [file Data_Sheet_1.PDF]

SUPPLEMENTARY MATERIALS

Evaluation of CONSRANK-like scoring functions for rescoring ensembles of protein-protein docking poses

Guillaume Launay[1,†], Masahito Ohue[2,†,*], Julia Prieto Santero[1], Yuri Matsuzaki[3], Cécile Hilpert[1], Nobuyuki Uchikoga[4], Takanori Hayashi[2], Juliette Martin[1*]

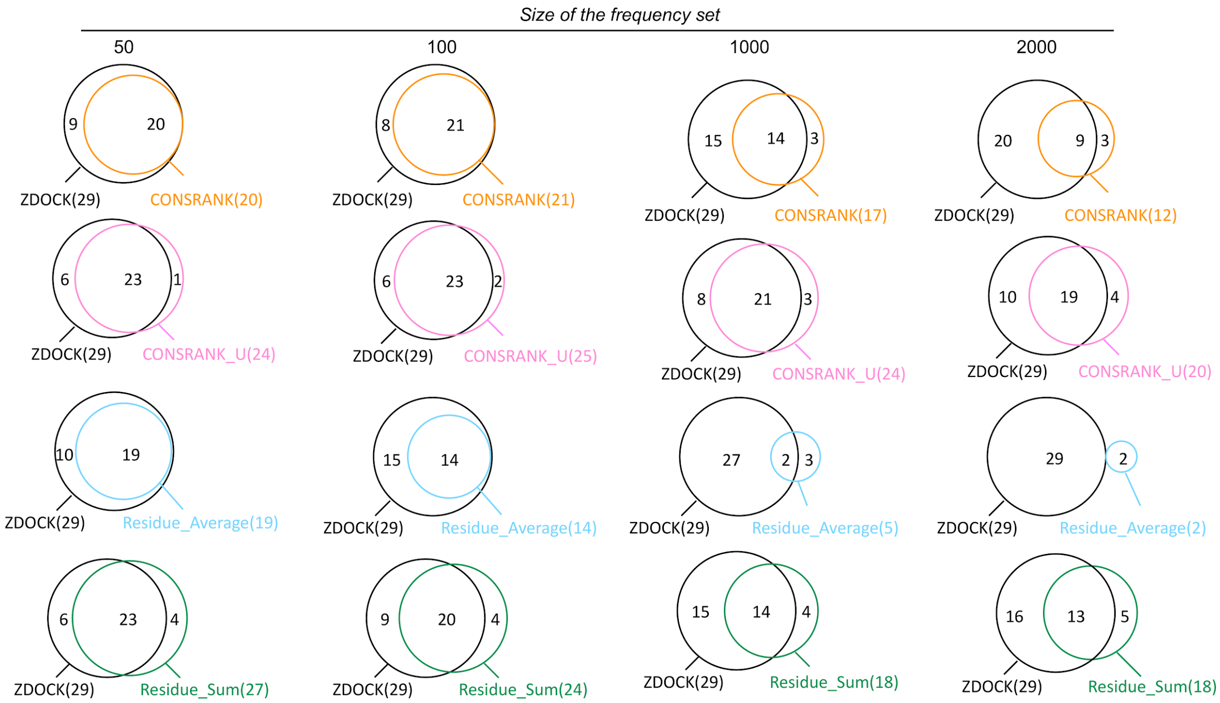

Figure S1. Venn diagrams showing the overlap between successful cases with the ZDOCK native scoring function and each of the rescoring functions.

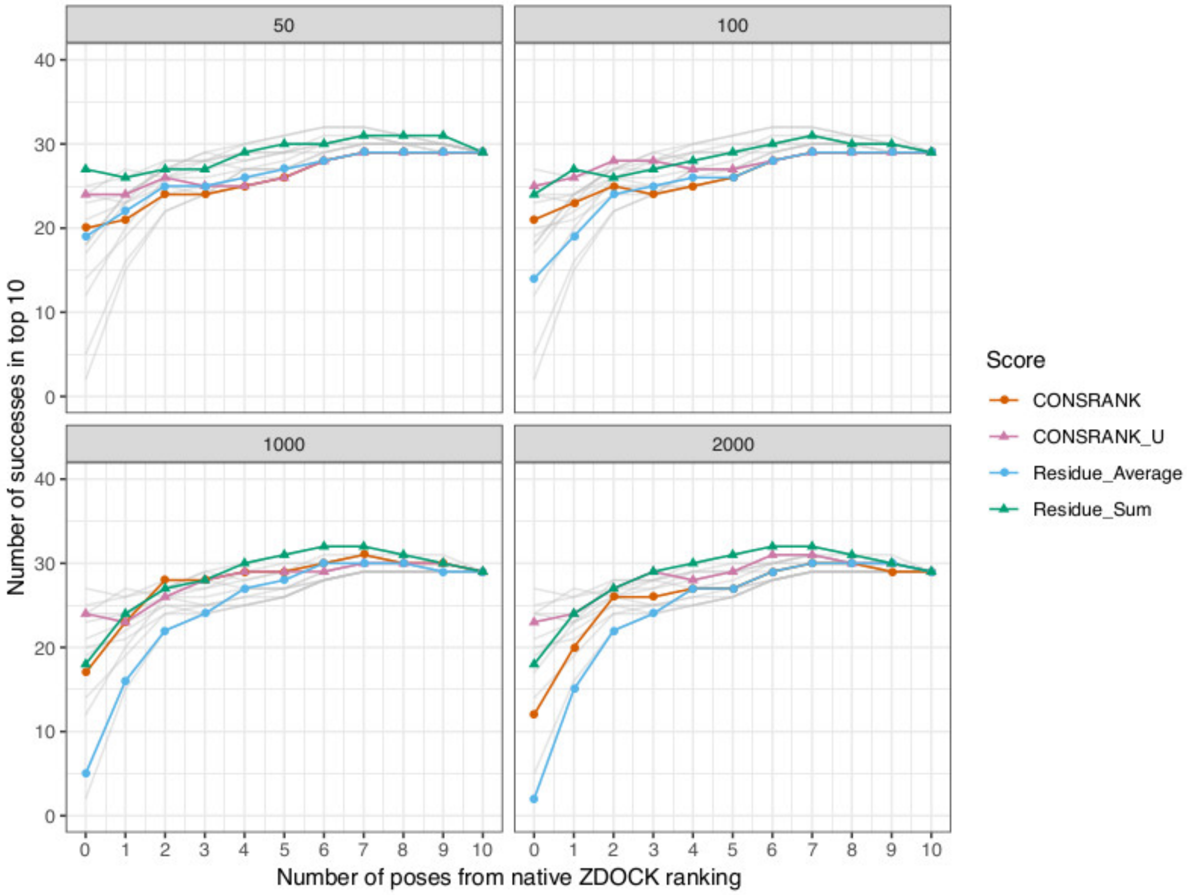

Figure S2. Number of successes after combination with the ZDOCK native scoring function. Each panel corresponds to different frequency sets, i.e., sets of poses used to compute the residue and contact scores from equations 1 and 4. In all cases, the first 2,000 solutions of ZDOCK are rescored. Grey lines represent data from other panels for comparison.
